# Dose escalation improves therapeutic outcome: post hoc analysis of data from a 12-week, multicentre, double-blind, parallel-group trial of trospium chloride in patients with urinary urge incontinence

**DOI:** 10.1186/1471-2490-10-15

**Published:** 2010-09-14

**Authors:** Rolf-Hasso Bödeker, Helmut Madersbacher, Claudia Neumeister, Michael Zellner

**Affiliations:** 1 Department of Statistics, Institute for Medical Informatics, University Clinic Giessen, Giessen, Germany; 2 Department of Neurology, University Hospital, Innsbruck, Austria; 3 Department of Medical Science/Clinical Research, Dr. R. Pfleger GmbH, Bamberg, Germany; 4 Johannesbad Specialist Clinic, Johannesstr. 2, D - 94072 Bad Füssing, Germany

## Abstract

**Background:**

Flexible dosing of anticholinergics used for overactive bladder (OAB) treatment is a useful strategy in clinical practice for achieving a maximum effective and maximum tolerated level of therapeutic benefit. In this post hoc analysis we evaluated the efficacy and tolerability of trospium chloride treatment for urinary urge incontinence (UUI) with focus on flexible dosing.

**Methods:**

The data came from a 12-week, randomised, double-blind, phase IIIb study in which 1658 patients with urinary frequency plus urge incontinence received trospium chloride 15 mg TID (n = 828) or 2.5 mg oxybutynin hydrochloride TID (n = 830). After four weeks, daily doses were doubled and not readjusted in 29.2% (242/828) of patients in the trospium group, and in 23.3% (193/830) in the oxybuytnin group, until the end of treatment. We assessed the absolute reduction in weekly UUI episodes and the change in intensity of dry mouth, recorded in patients' micturition diaries. Adverse events were also evaluated. Statistics were descriptive.

**Results:**

Dose escalation of either trospium or oxybutynin increased reduction in UUI episodes in the population studied. At study end, there were no relevant differences between the "dose adjustment" subgroups and the respective "no dose adjustment" subgroups (trospium: *P *= 0.249; oxybutynin: *P *= 0.349). After dose escalation, worsening of dry mouth was higher in both dose adjusted subgroups compared to the respective "no dose adjustment" subgroups (*P *< 0.001). Worsening of dry mouth was lower in the trospium groups than in the oxybutynin groups (*P *< 0.001). Adverse events were increased in the dose adjusted subgroups.

**Conclusions:**

Flexible dosing of trospium was proven to be as effective, but better tolerated as the officially approved adjusted dose of oxybutynin.

**Trial registration (parent study):**

The study was registered with the German Federal Institute for Drugs and Medical Devices (BfArM, Berlin, Germany), registration number 4022383, as required at the time point of planning this study.

## Background

Antimuscarinics (e.g., trospium chloride, tolterodine, darifenacin, oxybutynin) are effective in reducing detrusor overactivity and hence urgency and urinary urge incontinence (UUI). However, by virtue of their mechanism of action the tolerability and usefulness of these drugs is often diminished by relatively common and well-known peripheral side effects. Optimal treatment should therefore be individualised, considering the patient's co-morbidities, concomitant medications and the pharmacological profiles of the different drugs [1-6].

Flexible dosing of anticholinergics is a common and useful strategy in clinical practice for achieving a maximum effective and maximum tolerated level of therapeutic benefit [7-9]. Patients who are most responsive to the drug can therefore receive the lowest effective dose; other patients could achieve greater improvements in symptoms and quality of life with a tailor-made dose adjustment. As most patients with overactive bladder (OAB) symptoms are elderly (65 years or older), considerations for safety and compliance are of special importance. Although dose escalation can be implemented with nearly all of the anticholinergics, it is done most often and approved with oxybutynin [9,10]. In the case of trospium chloride (TC) urologists prescribe the drug in other, the recommended daily dosage of 45 mg often exceeding doses for treatment of UUI/OAB [11], especially in patients with neurogenic detrusor overactivity [2,12]. However, to date dose escalation of individual daily medication is not mentioned in the SPC of trospium.

The noninferiority of trospium 45 mg per day in comparison to oxybutynin (OXY) 7.5 mg per day exploring efficacy, tolerability and effects on health-related quality of life parameters has previously been assessed in patients with documented urinary frequency plus urge incontinence in a large, 12-week, randomised, double-blind, flexible-dose phase IIIb trial [13]. Daily dosages could be increased upward after four weeks, to 90 mg of trospium and 15 mg of oxybutynin, respectively, if needed. The purpose of the present post hoc analysis was to compare the effects and the tolerability of flexible dosing in those patients stratified according to "dose adjustment" and "no dose adjustment" subgroups. The objective was not to evaluate "data driven" statistically significant results, but to reveal detailed advice and answers to the results of the study.

## Methods

This is a post hoc analysis of data from a randomised, double-blind, active-controlled, parallel-group, flexible-dose phase IIIb trial conducted at 153 urological centres in Germany; details of the study design and methods were published previously [13]. The study was carried out in accordance with the German Drug Law, the Revised Declaration of Helsinki, and the European Union standards of Good Clinical Practice. The independent ethics committee of the Bavarian State Chamber of Physicians (Munich, Germany) approved the study protocol, the protocol amendment, the information sheet, and the consent form before the start of the study (reference number: 04075; Munich, 15 July 2004).

### Patients

Eligibility for the study was determined by the completion of a 7-day micturition diary before randomisation. Men and women (18 years of age or older) with urinary frequency (eight or more micturitions every 24 hours) plus urge incontinence (five or more episodes per week) met the inclusion criteria.

Based on this first diary, subjects with a total daily urine volume of 2.8 L or more, a mean micturition volume of more than 250 mL, and/or a clinically significant bladder outlet obstruction (i.e., postvoid residual urine volume of more than 100 mL) were excluded from participation, as were those with an indwelling catheter or intermittent self-catheterization; urinary tract infection at the screening visit; interstitial cystitis and/or hematuria; contraindications to anticholinergic therapy (e.g., untreated narrow-angle glaucoma, mechanical gastrointestinal stenosis, myasthenia gravis syndrome), tachycardiac arrhythmia, severe psychiatric illnesses, hypersensitivity to trospium or oxybutynin or one of the vehicle ingredients; participation in a bladder training or electrostimulation program, or in another study within the past 30 days. Patients currently receiving drug treatment for UUI were allowed to enter the study following a 14-day washout period prior to study treatment. Concomitant treatments (i.e., other anticholinergic drugs or drugs possessing significant anticholinergic or sympathomimetic effects) as well as drugs that could interact with trospium or oxybutynin were prohibited at all times during the study. However, α-adrenergic blockers were permitted.

### Study Design

Having given their written informed consent to the study, patients were randomised on a computer-generated block design ratio (1:1) stratified by centre at the entrance visit to receive 15 mg trospium chloride three times a day (TID) or 2.5 mg oxybutynin hydrochloride TID for 12 weeks. On the basis of the symptoms recorded in a second micturition diary and on the investigator's and patient's impression of the individual's tolerance of the study medication, daily doses could be adjusted upward after four weeks of treatment (at the intermediate visit), to 90 mg trospium (30 mg TID) or 15 mg oxybutynin (5 mg TID) for the following eight weeks. In the case of adverse events (AEs), the increased dosage could be readjusted to the starting point after one week. Patients whose symptoms were managed with the standard dose continued to receive the starting dosage of medication throughout the study. The study ended for each patient after 12 weeks of treatment (final visit).

### Efficacy and tolerability assessment

For the post hoc analysis, patients in both treatment groups were stratified to subgroups according to "dose adjustment" or "no dose adjustment".

The primary efficacy variable for the current analysis - as for the parent study - was the absolute reduction in weekly UUI episodes comparing baseline values (i.e., the last week before the entrance visit) with those of treatment week 12 (i.e., the last week before the final visit). This quantitative symptom change was calculated from the patients' 7-day micturition diaries, which had been completed by each patient before randomisation and at treatment week 4 and 12. The intensity of dry mouth, one of the secondary efficacy variables in the parent study, was recorded on an ordinal scale (*none*, *mild*, *moderate*, or *severe*) and also evaluated from the entrance visit to the intermediate and final visits.

Tolerability was assessed by evaluating the spontaneously reported adverse events, and discontinuations in the subgroups. The association between AEs and treatment was based on physician-assessed causality.

All other efficacy and tolerability assessments conducted in the parent study were described there [13].

### Statistical Analysis

The post hoc inferential analysis was performed using the full analysis set (FAS) of the parent study comprising all participants who belonged to the safety population (all patients who were exposed to study medication at least once) and for whom any post-randomisation efficacy data were available. All statistical analyses were descriptive. As normal distribution of the parameters of interest could not be assumed for between-group comparisons, we used the Wilcoxon rank sum test (van Elteren procedure), stratified by study centre for continuous variables. The Hodges-Lehmann point estimator (HL_est_) was used as a nonparametric estimator. Interval estimates were provided in the form of nonparametric 2-sided 95% CI. The distributions of the observed data are presented by median, quartiles, minimum, and maximum.

## Results

The population of the current analysis, which corresponds to the safety population of the parent study, included 828 patients (49.9%) randomised to trospium chloride 45 mg/d and 830 patients (50.1%) randomised to oxybutynin 7.5 mg/d at 153 urological centres in Germany. After four weeks, 29.2% (484 of 1658) had their dose adjusted to the permitted increased (i.e., doubled) dose: 31.5% (261 of 828) of patients in the trospium group, and in 26.9% (223 of 830) of patients in the oxybutynin group. After one week, however, 10.1% (49 of 484) of them (19 in the trospium group and 30 in the oxybuytnin group) had subsequent dose readjustments that returned them to the standard starting dose until the end of treatment.

The FAS population comprised 1608 patients (TC: 810; OXY: 798). No post-randomisation data were available for 50 patients: 18 (2.2%) in the TC-group and 32 (3.9%) in the OXY-group. Patient demographics revealed no clinically relevant differences between the treatment groups. The mean (SD) age of patients was 61.3 (12.17) years (median: 63 years; range: 20-91 years), 90.3% (n = 1452) were women. Other patient demographics and characteristics were previously reported [13].

### Urge incontinence episodes

Treatment with either trospium chloride or oxybutynin reduced the total number of UUI episodes per week in the patient population selected by the defined in- and exclusion criteria. Table 1 lists the results of the patients' diaries.

**Table 1:** Changes in UUI episodes per week by dose adjustment in the TC-treatment group and the OXY- treatment group (FAS)

	Trospium chloride			Oxybutynin		
Time point	No Dose Adjustment	Dose Adjustment	Difference between subgroups	No Dose Adjustment	Dose Adjustment	Difference between subgroups
*Entrance visit (baseline)*						
N	546	240		575	192	
Mean ± SD	20.1 ± 17.6	22.2 ± 19.58		18.6 ± 16.37	20.8 ± 18.41	
Median	14.0	15.0		14.0	13.0	
Range	0.0 - 107.0	4.0 - 99.0		0.0 - 120.0	0.0 - 90.0	
*Intermediate visit*						
N	531	241		561	193	
Mean ± SD	7.9 ± 11.97	12.2 ± 16.4		6.5 ± 10.32	13.4 ± 17.68	
Median	3.0	6.0		3.0	6.0	
Range	0.0 - 67.0	0.0 - 112.0		0.0 - 109.0	0.0 - 83.0	
*Final visit*						
N	502	226		534	181	
Mean ± SD	5.4 ± 10.42	7.8 ± 12.49		4.4 ± 9.28	6.9 ± 13.42	
Median	1.0	3.0		0.0	1.0	
Range	0.0 - 64.0	0.0 - 65.0		0.0 - 80.0	0.0 - 100.0	
*Change from baseline to final visit*						
N	508	228		537	182	
Mean ± SD	-14.96 ± 15.063	-14.40 ± 16.346		-14.29 ± 15.673	-13.56 ± 15.982	
Median	-11.00	-10.58		-10.00	-9.00	
Range	-107.0 - 28.0	-96.0 - 22.0		-105.0 - 63.0	-88.0 - 50.0	
Hodges-Lehmann estimate			-1.00			-1.00
95% CI (non-parametric)			[-2.00; 0.00]			[-2.00; 1.00]
*P*-value			0.248989			0.349402

Note: *P*-values are obtained from the generalised Wilcoxon Rank sum test without stratification for centre.

**Figure 1:**
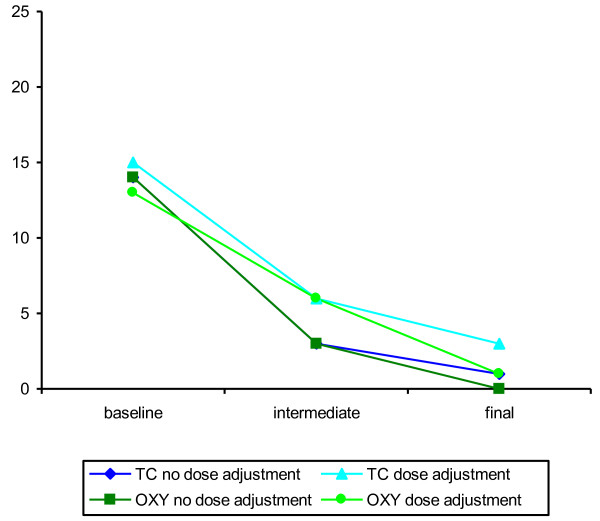
**Changes in UUI episodes per week by dose adjustment (median values, FAS)**.

In the FAS set, the baseline values of UUI episodes per week (median and mean values) of the "dose adjustment" subgroups were slightly higher than the baseline values of the "no dose adjustment" subgroups in both treatment groups, with the only exception concerning the baseline median values in the oxybutynin "dose adjustment" subgroup. Until the intermediate visit all patients received the standard dose of the relevant medication. At the intermediate visit the patients who fulfilled the criteria for dose adjustment still suffered from a higher number of weekly UUI episodes compared to those patients who needed no dose adjustment. At the final visit the absolute changes in weekly UUI episodes from baseline to week 12 (primary endpoint) showed no relevant differences between the "dose adjustment" subgroups and the "no dose adjustment" subgroups in both treatment groups. Figure 1 illustrates median changes in weekly UUI episodes at each visit for both treatment groups and all subgroups. In the FAS the median change was -11.00 in the "no dose adjustment" TC-subgroup and -10.58 in the "dose adjustment" TC-subgroup; in the oxybutynin treatment group the median change was -10.00 in the "no dose adjustment" subgroup and -9.00 in the "dose adjustment" subgroup. There was no indication whatsoever of a significant difference between the subgroups ("no dose adjustment" and "dose adjustment") in terms of this change neither in the trospium group (*P *= 0.249) nor in the oxybutynin group (*P *= 0.349). This is also obvious from the corresponding non-parametric estimator (HL_est_) for the difference between both subgroups and the corresponding 95% CI.

### Intensity of mouth dryness

The percentage of patients without mouth dryness was comparable between the treatment groups at baseline (trospium: 46.8% [257 of 549; "no dose adjustment"]/49.2% [119 of 242; "dose adjustment"] versus oxybutynin: 45.6% [262 of 575]/45.6% [88 of 193]). At the final visit, the percentage of patients reporting no dry mouth was 19.5% (107 of 549) in the "no dose adjustment" TC-subgroup versus 13.2% (32 of 242) in the "dose adjustment" TC-subgroup, and 8.7% (50 of 575) in the "no dose adjustment" OXY-subgroup versus 4.7% (9 of 193) in the "dose adjustment" OXY-subgroup.

**Figure 2:**
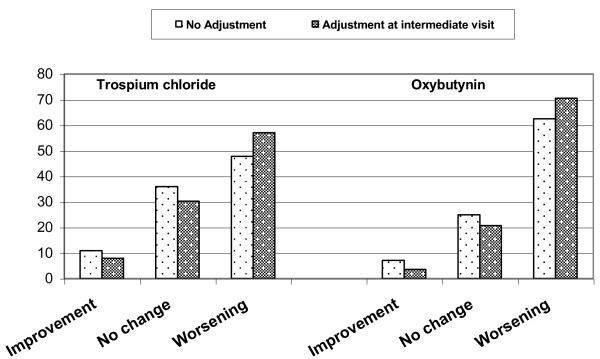
**Changes in intensity of dry mouth by dose adjustment (%, FAS)**.

At week 4 and 12, the increase in "dry mouth" intensity was significantly lower (*P *< 0.001) in the trospium groups than in the oxybutynin groups. The percentage of patients who experienced worsening of dry mouth from baseline to end of study was 57.0% (138 of 242) in the "dose adjustment" TC-subgroup versus 70.5% (136 of 193) in the "dose adjustment" OXY-subgroup, and 47.7% (262 of 549) in the "no dose adjustment" TC-subgroup versus 62.4% (359 of 575) in the "no dose adjustment" OXY-subgroup (Figure 2). Thus, patients of both dose adjusted subgroups experienced an approximately 10% higher rate of worsening of mouth dryness than patients of the respective "no dose adjustment" groups at the end of study. The differences between the subgroups were significant for the time period between the intermediate (i.e., time of dose escalation) and the final visit (worsening for TC: 43.4% [105 of 242] in the "dose adjustment" subgroup versus 20.0% [110 of 549] in the "no dose adjustment" subgroup, *P *< 0.001; OXY: 43.5% [84 of 193] versus 19.5% [112 of 575], *P *< 0.001), whereas the analysis of the changes from baseline to the end of treatment revealed no significant difference between the "dose adjustment" and the "no dose adjustment" subgroups for both treatment groups (TC: *P *= 0.049; OXY: *P *= 0.071).

### Tolerability

The safety population of the parent study comprised 1658 patients. The tolerability assessments of the current analysis based on 1609 patients, i.e., all patients of the safety population minus those patients (n = 49) who had dose readjustment because of AEs (19 [2.3%] in the trospium group and 30 [3.6%] in the oxybutynin group). The main reasons for dose readjustment that were determined to be definitely, probably, or possibly treatment-related were gastrointestinal disorders, such as dry mouth (n = 24), nausea (n = 7), and constipation (n = 3). More patients (n = 17) treated with oxybutynin 15 mg/d experienced the most frequent disorder dry mouth than those receiving trospium 90 mg/d (n = 7). Most cases of dry mouth were moderate to severe.

Regarding the time period between the intermediate visit (i.e., the time of dose escalation) and the final visit, treatment related AEs occurred in 43 out of 809 patients (5.3%) taking trospium and in 58 out of 800 patients (7.3%) taking oxybutynin. Hereby, patients of both dose adjusted subgroups suffered from a slightly higher rate of AEs possibly related to the study medication than those of the "no dose adjustment" subgroups (trospium: 9.5% [23 of 242] versus 3.5% [20 of 567], *P *< 0.001; oxybutynin: 10.9% [21 of 193] versus 6.1% [37 of 607], *P *= 0.026). However, this fact did not lead to a higher rate of study discontinuations in the dose adjusted subgroups, neither in the TC-subgroup (5.4% [13 of 242] in the "dose adjustment" subgroup versus 5.1% [29 of 567] in the "no dose adjustment" subgroup, *P *= 0.880) nor in the OXY-subgroup (6.2% [12 of 193] versus 6.1% [37 of 607], *P *= 0.951) regarding the same time interval.

Treatment-related AEs were typically anticholinergic in nature, including dry mouth, constipation, nausea, dyspepsia, and diarrhoea. Full details of the tolerability assessments based on the data from the parent study were described previously [13].

## Discussion

This post hoc analysis based on the first randomised, controlled, double-blind study performed to evaluate the use of flexible-dose regimen of trospium chloride in patients with UUI [13]. The current analysis of bladder diary variables from the parent phase IIIb trial shows that dose escalation of either trospium or oxybutynin increased reduction in UUI episodes in the randomised patient population studied. At the intermediate visit the patients who received dose adjustment still had suffered from a higher number of weekly UUI episodes compared to the "no dose adjustment" subgroups. This indicates that there are really a certain proportion of OAB-patients (approximately 30% in the trospium group) who might profit from a higher dosage for a better treatment success. At the end of study, no relevant differences in treatment success between the dose adjusted and the "no dose adjustment" subgroups could be found, indicating that the total number of weekly UUI episodes was indeed more reduced in patients with dose escalation by the end of treatment than in patients treated with the standard dosage.

The standard doses of trospium and oxybutynin applied in this study are defined in the respective Summary of Product Characteristics (SPC) and are commonly used in general practice. However, the flexible dosing strategy allowed in this study reflects real-life use [11], and permits treatment to be tailored to patients' individual needs based on their individual responses to the drug. To approximate clinical practice, the flexible dosing design of the study allowed the investigator together with the patient to decide whether to increase the dose or to remain the starting dose by the end of treatment. This decision was based on the patient's micturition diary and the subjective feeling of side effects. Only 29.2% of patients (484 of 1658) required increased doses after four treatment weeks. This suggests that the recommended dosage regimens are adequate for most patients with OAB. On the other hand, it shows that dose escalation can really improve the therapeutic outcome of anticholinergic therapy. The flexible-dosing design of the parent study therefore reflects how OAB might be successfully managed in clinical practice. Optimal treatment of OAB should be individualised, considering the patient's comorbidities and concomitant medications, and the pharmacological profiles of the different drugs, as recommended in international guidelines for OAB therapy [1-4]. The goal of UUI therapy is simply to get the patient as dry as possible. If the primary care physician has the possibility of flexible dosing to facilitate meeting the patient's goal, it offers him a real chance of greater improvements in patient's symptoms and quality of life.

Adjusting dosage is indeed a common practice for several anticholinergic drugs [6-10]. Recent studies with extended-release oxybutynin [14-17], darifenacin [18], solifenacin [19], and fesoterodine [20,21] suggest that reduction of urinary urge and total incontinence episodes may be improved significantly with a flexible dosing strategy.

Improved efficacy associated with dose escalation has also been demonstrated in patients with neurogenic detrusor overactivity [22] treated with trospium chloride [12] or oxybutynin [23,24].

However, to optimize the therapeutic index, improvements in efficacy by using flexible dosing must be balanced by gains in safety and tolerability. By virtue of their mechanism of action, antimuscarinics commonly interact with muscarinic receptors throughout the body, thereby affecting a variety of physiological functions [25]. A somewhat higher rate of AEs and mouth dryness in the dose adjusted subgroups of both treatment arms could be detected only by the inferential analysis of the present study. However, both events did not lead to a higher rate of study discontinuations, neither in the trospium nor in the oxybutynin subgroups. The results of the above mentioned dose titration studies support this finding, indicating that patients are willing to tolerate a certain degree of dry mouth and AEs to reach the optimal balance between continence and side effects [8,9,21].

Regarding the rate of AEs in the subgroups with adjusted daily doses it is remarkable that the adjusted daily dose of OXY corresponds to the recommended maximum daily dose approved by the regulatory authorities.

## Conclusions

This post hoc analysis evaluating the efficacy and tolerability of trospium chloride demonstrated that urinary frequency and urge incontinence can be reduced significantly with a flexible dosing strategy. Dose adjustment may improve the therapeutic outcome in some patients, facilitating the definite balance between the efficacy and anticholinergic side effects such as dry mouth. In comparison to the adjusted dose of oxybutynin which is officially approved, the adjusted dose of trospium was proven to be at least as well tolerated, safe and efficient.

## Competing Interests

Dr. R. Pfleger GmbH (Bamberg, Germany) sponsored the parent study and the post hoc analysis. RHB is paid consultant to Dr. R. Pfleger GmbH. CN is Project Manager Clinical Research of Dr. R. Pfleger GmbH. HM and MZ declare that they have no competing interests to disclose.

## Authors' contributions

MZ was the principal investigator of the parent study. HM was member of the study's independent advisory board. CN coordinated the study and the external monitoring. RHB performed the statistical planning and supervised the statistical analysis of the parent study and the post hoc analysis as well. All authors were responsible for critically revising the manuscript. All authors have given final approval of this version for publication.

## Pre-publication history

The pre-publication history for this paper can be accessed here:

http://www.biomedcentral.com/1471-2490/10/15/prepub
